# Saline Treatment of Acute Dysentery

**Published:** 1864-08

**Authors:** Jas. Bolton

**Affiliations:** Surgeon P. A. C. S.


ART. VI.—*Saline Treatment of Acute Dysentery.* By JAS. BOLTON, Surgeon P. A. C. S.

Any one who has had to deal with this disease, especially as it infests camps, must have been much annoyed by its refractory character. Calomel and opium, found so efficacious in diarrhœa, may have produced their full effect; and the evacuations may have been so greatly improved, that the physician will congratulate himself that he has obtained a complete mastery of the disease, and yet the very next evacuation will dash all his hopes. The characteristic bloody mucus may return, accompanied by tormina and tenesmus in full force. Calomel may be pushed to salivation, and yet the disease will be unsubdued. Purgatives are of great value, but of these, my experience is greatly in favor of the salines.

I give the patient immediately the following: hydrarg chlor. mit.; pulv. ipecac and opii aa, 10 to 15 grs.; or, if the stomach be irritable, ¼ gr. to ½ gr. of a salt of morphia, instead of the latter ingredient. After an interval of three hours, I commence with the following mixture: magnes. sulphat., 1 oz.; tinct. opii, 1 dr.; aq. menth. pip., q. s.—to make a four-ounce mixture, s. a.; tablespoonful every third hour, until two or three copious watery evacuations shall have been produced. After this, opium may be required to restrain the action of the bowels, and to relieve pain. Usually, after the action of the saline shall have been fully established, the disease is at an end; and this is frequently accomplished in thirty-six hours. If the attack prove obstinate, the calomel will have to be repeated, and blisterings, opiate enemata, &c., may be required.

Sometimes there is a malarial element superadded, as evinced by periodicity of fever, and then it will be necessary to interpose quinia.

*Rationale.*—The calomel, by its action upon the liver, emulges the portal vessels, and thus relieves the hyperœmic condition of the mesenteric vessels. This action is seconded by the saline cathartic, which produces copious serous evacuations, and thus produces local depletion.

The contraction of the distended vessels, and washing away of the adherent mucus, remove the sensation as of a foreign body to be discharged, and thus relieve the distressing tenesmus.

After an extensive employment of this practice during many years, I can testify that I scarcely know any more satisfactory method of treatment in the whole range of medical practice, than the saline treatment of acute dysentery.

